# Barriers and Unmet Educational Needs Regarding Implementation of Medication Adherence Management Across Europe: Insights from COST Action ENABLE

**DOI:** 10.1007/s11606-024-08851-2

**Published:** 2024-06-28

**Authors:** Gaye Hafez, Emma Aarnio, Sara Mucherino, Maria Kamusheva, Miriam Qvarnström, Ines Potočnjak, Indre Trečiokiene, Jovan Mihajlović, Marie Ekenberg, Job F. M. van Boven, Francisca Leiva-Fernández, European Network to Advance Best Practices Technology on Medication AdherencE (ENABLE)

**Affiliations:** 1 Department of Pharmacology, Faculty of Pharmacy, Altinbas University, Istanbul, Turkey; 2 School of Pharmacy, University of Eastern Finland, Kuopio, Finland; 3 Federico II University of Naples, Naples, NA Italy; 4 Faculty of Pharmacy, Medical University of Sofia, Sofia, Bulgaria; 5 Department of Pharmacy, Faculty of Pharmacy, Uppsala University, Uppsala, Sweden; 6 Sestre Milosrdnice University Hospital Center, School of Medicine Catholic University of Croatia, Zagreb, Croatia; 7 Faculty of Medicine, Vilnius University, Vilnius, Lithuania; 8 Mihajlović Health Analytics, Novi Sad, Serbia; 9 University of Novi Sad, Medical Faculty, Novi Sad, Serbia; 10 Department of Clinical Pharmacy and Pharmacology, Medication Adherence Expertise Center of the Northern Netherlands (MAECON), University Medical Center Groningen, University of Groningen, Groningen, Netherlands; 11 Andalusian Health Service-Málaga-Guadalhorce Health District-IBIMA-Platform BIONAND-University of Malaga, Malaga, Spain

**Keywords:** medication adherence, unmet needs, training, barriers, healthcare

## Abstract

**Background:**

Medication adherence is essential for the achievement of therapeutic goals. Yet, the World Health Organization estimates that 50% of patients are nonadherent to medication and this has been associated with 125 billion euros and 200,000 deaths in Europe annually.

**Objective:**

This study aimed to unravel barriers and unmet training needs regarding medication adherence management across Europe.

**Design:**

A cross-sectional study was conducted through an online survey. The final survey contained 19 close-ended questions.

**Participants:**

The survey content was informed by 140 global medication adherence experts from clinical, academic, governmental, and patient associations. The final survey targeted healthcare professionals (HCPs) across 39 European countries.

**Main Measures:**

Our measures were barriers and unmet training needs for the management of medication adherence across Europe.

**Key Results:**

In total, 2875 HCPs (pharmacists, 40%; physicians, 37%; nurses, 17%) from 37 countries participated. The largest barriers to adequate medication adherence management were lack of patient awareness (66%), lack of HCP time (44%), lack of electronic solutions (e.g., access to integrated databases and uniformity of data available) (42%), and lack of collaboration and communication between HCPs (41%). Almost all HCPs pointed out the need for educational training on medication adherence management.

**Conclusions:**

These findings highlight the importance of addressing medication adherence barriers at different levels, from patient awareness to health system technology and to fostering collaboration between HCPs. To optimize patient and economic outcomes from prescribed medication, prerequisites include adequate HCP training as well as further development of digital solutions and shared health data infrastructures across Europe.

**Supplementary Information:**

The online version contains supplementary material available at 10.1007/s11606-024-08851-2.

## INTRODUCTION

We live in an era with highly effective medications available. However, medication adherence (MA) is a key prerequisite for these medications to work adequately. MA is the degree to which patients take their medications as recommended by their healthcare providers.^1^ Suboptimal adherence rates are associated with an increased number of consultations, increased rates and duration of hospital stays, and higher costs for the healthcare system and society.^2^ Indeed, the Organisation for Economic Co-operation and Development (OECD) estimated that medication nonadherence in Europe alone is associated with 125 billion euros and 200,000 deaths on an annual basis.^3^ Similarly, in the United States (USA), the economic impact of nonoptimized drug therapy is estimated at 500 billion USD.^4^ Despite these consequences, adherence rates in daily clinical practice are suboptimal. The World Health Organization (WHO) report estimated that only 50% of patients adhere to long-term therapy.^1^ Since 2003, many reasons for non-adherence have been identified across a range of therapeutic areas and countries.^5–7^ Notably, these reasons can be situated at the patient (e.g., beliefs, cognition, comorbidities, knowledge), treatment (e.g., side effects, dosing regimen, co-medication), and/or health system level (e.g., access to medication, communication with healthcare providers, social and information technology [IT] support).^8–11^ To address nonadherence, different interventions have been tested showing varying results ^12,13^ with the vast majority focusing on a single factor related to nonadherence.^14^ Most of these interventions have targeted either a patient-level barrier (e.g., sending electronic reminders) or a treatment-level barrier (e.g., reducing the dosing regimen).^15^ Much less is known about health system barriers to support MA and its management by healthcare professionals (HCPs). In a qualitative USA study, HCPs and patients emphasized that communication difficulties can impact MA.^16^ Continuity of care was identified as an important prerequisite for MA by Irish HCPs.^17^ A small study among 16 general practitioners in Finland identified enhanced coordination of care, improved patient education and IT systems, and more interprofessional involvement in patients’ follow-up as key barriers, yet whether these results can be extrapolated across European countries and HCPs is unknown.^6^ European-wide surveys have highlighted that only half of HCPs inquire about adherence with their patients ^18^, yet a broad understanding of HCP perceived barriers to adequate MA management in Europe is still lacking.

The European Cooperation in Science and Technology (COST) project “European Network to Advance Best practices & technoLogy on medication adherence” (ENABLE) with members from 40 European countries aims to raise awareness of adherence-enhancing solutions and to foster and extend multidisciplinary knowledge at patient, treatment, and system levels.^19^ This study aimed to identify HCP-perceived health system barriers and unmet needs regarding MA management in Europe.

## METHODS

### Study Design

A cross-sectional study was conducted through an online survey. This study was reported according to the CHERRIES checklist for online surveys (Supp Table S1).^20^

### Survey Design

The design of this study was informed by an open-ended pre-survey (Supp Table S2) among 142 MA experts residing in one of the 39 ENABLE countries (of which 140, i.e., 98.6%, from 35 different countries responded) from health, academic, governmental, and patient associations with typically four to five experts/country (min, 1; max, 8) (Supp Table S3). Experts were selected by ENABLE country representatives based on their clinical, policy, and/or research expertise. Most experts (*n* = 74; 52.9%) represented a research/academic organization, followed by the hospital (*n* = 39; 27.9%) and primary care (*n* = 27; 19.3%) settings (Supp Table S4). The distribution was similar between the three European regions, except for hospital respondents, which were lower in the Eastern (18.9%) and higher in the Western European regions (34.4%). Experts were asked about barriers and unmet needs in their country. An ENABLE group analyzed their responses (Supp Table S5) using the framework method.^21^ The qualitative results (Supp Table S6, Figure S1) were turned into a final HCP survey containing 19 close-ended questions (Supp Table S7a, S7b).

### Survey Administration

The survey was open from July to November 2022. Due to country-specific differences, the mode of distribution of the survey was decided by ENABLE representatives and different methods (e.g., connections through health institutions, associations, forums, personal contacts, networks of HCPs, and official e-mails) were used to achieve the largest sample possible. The surveys were created using the online Webropol 3.0 survey and reporting tool (https://webropol.com/) and piloted before large-scale administration. The survey was voluntary, anonymous and collected no personal information. No incentives were provided to participants. The survey was available in English and 24 other European languages.

### Measures

The two main measures of this study were (i) the HCP perceived barriers to MA management and (ii) HCP training needs regarding MA management.

### Analyses

We conducted descriptive analyses on HCP survey data, stratified by physicians, pharmacists, and nurses. Geographical variations were examined using the latest OECD classification for Europe (Western, Central, and Eastern) based on the Global Burden of Disease (GBD) study. Gradual colour codings depicted the relative importance of barriers or training needs, ranging from dark green (highest) to pink (lowest). All analyses were performed using SPSS software version 27.

### Ethics

The study was approved by the Research Ethics Committee of Malaga, Spain (Number 1932/ 29–04-2021), Croatia (Number 501–04/01–06-2021; Number 251–29-11–22-05/08–09-2022), the Republic of North Macedonia (Number 2005–133/3/ 06–05-2021), and Turkey (Number 24714/16–02-2022). In other countries, no formal approval was needed according to local legislation. The study was conducted by the principles established in the Declaration of Helsinki, the Council of Europe Convention on Human Rights and Biomedicine, and the requirements established in each ENABLE country legislation. The study conformed to the norms of good clinical practice (art. 34 RD 223/2004; Community Directive 2001/20/CE) and the provisions of Regulation 2016/679 of the European Parliament and of the Council of April 27, 2016, on Data Protection (GDPR).

All participants provided online informed consent before starting the survey.

## RESULTS

### Participants

All participants answered the background questions. The informed consent rate was 98% and only one participant did not complete the survey which was not included. A total of 2875 HCPs from 37 countries replied to the HCP survey, predominantly pharmacists (40%), physicians (37%), and nurses (16%) (Table 1). Of the 2875 respondents, 1049 (36%) were from Western, 1351 (47%) were from Central, and 475 (17%) were from Eastern Europe. Most responses (*N* = 432) came from Romania and the lowest number of responses (*N* = 1) came from Luxembourg and Norway (Supp Table S8). Most of the respondents (33%) had more than 20 years of working experience. No responses were received from Czechia, Denmark, and Moldova (Supp Table S8).

**Table 1:** Characteristics of the respondents (*N* = 2875)

	**Count (%)**	**Count (% within European Region)**			
	**Europe**	**Western Europe**	**Central Europe**	**Eastern Europe**	***p*-value**
**Respondents**	2875 (100)	1351 (47.0)	475 (16.5)	1049 (36.5)	
**Profession**					0.000
Pharmacist	1148 (39.9)	761 (56.3)	103 (21.7)	284 (27.1)	
Physician	1055 (36.7)	440 (32.6)	245 (51.6)	370 (35.3)	
Nurse	473 (16.4)	104 (7.7)	56 (11.8)	313 (29.8)	
Dentist	51 (1.8)	27 (2.0)	15 (3.2)	9 (0.9)	
Midwife	38 (1.3)	0	36 (7.6)	2 (0.2)	
Other	110 (3.8)	19 (1.4)	20 (4.2)	71 (6.80)	
**Workplace**					0.000
Community pharmacy	988 (34.4)	705 (52.2)	97 (20.4)	186 (17.7)	
Hospital	824 (28.7)	284 (21.0)	207 (43.6)	333 (31.7)	
Primary care	778 (27.1)	261 (19.3)	143 (30.1)	374 (35.7)	
Home care	50 (1.7)	3 (0.2)	6 (1.3)	41 (3.9)	
Hospital pharmacy	49 (1.7)	22 (1.6)	3 (0.6)	24 (2.3)	
Nursing home	33 (1.1)	3 (0.2)	1 (0.2)	29 (2.8)	
Hospice	8 (0.3)	4 (0.3)	0	4 (0.4)	
Other	145 (5.0)	69 (5.1)	18 (3.8)	58 (5.5)	
**Working area**					0.000
Urban	2231 (77.6)	1100 (81.4)	413 (86.9)	718 (68.4)	
Rural	366 (12.7)	134 (9.9)	44 (9.3)	188 (17.9)	
Both	278 (9.7)	117 (8.7)	18 (3.8)	143 (13.6)	
**Working sector**					0.000
Public	1812 (63.0)	742 (54.9)	343 (72.2)	727 (69.3)	
Private	1022 (35.5)	601 (44.5)	129 (27.2)	292 (27.8)	
Third/civic	41 (1.4)	8 (0.6)	3 (0.6)	30 (2.9)	
**Overall work experience**					0.000
Less than 5 years	621 (21.6)	302 (22.4)	125 (26.3)	194 (18.5)	
5–9 years	508 (17.7)	264 (19.5)	112 (23.6)	132 (12.6)	
10–20 years	788 (27.4)	429 (31.8)	105 (22.1)	254 (24.2)	
More than 20 years	958 (33.3)	356 (26.4)	133 (28.0)	469 (44.7)	

### Barriers to HCP MA Management

In Europe, the largest patient barrier regarding MA management in HCPs’ daily work was the “lack of awareness among patients” (mentioned by 65.9% of HCPs) (Table 2). This referred to patients not being aware of the importance of adherent drug intake and was consistently ranked highest across all three European regions.

**Table 2.**
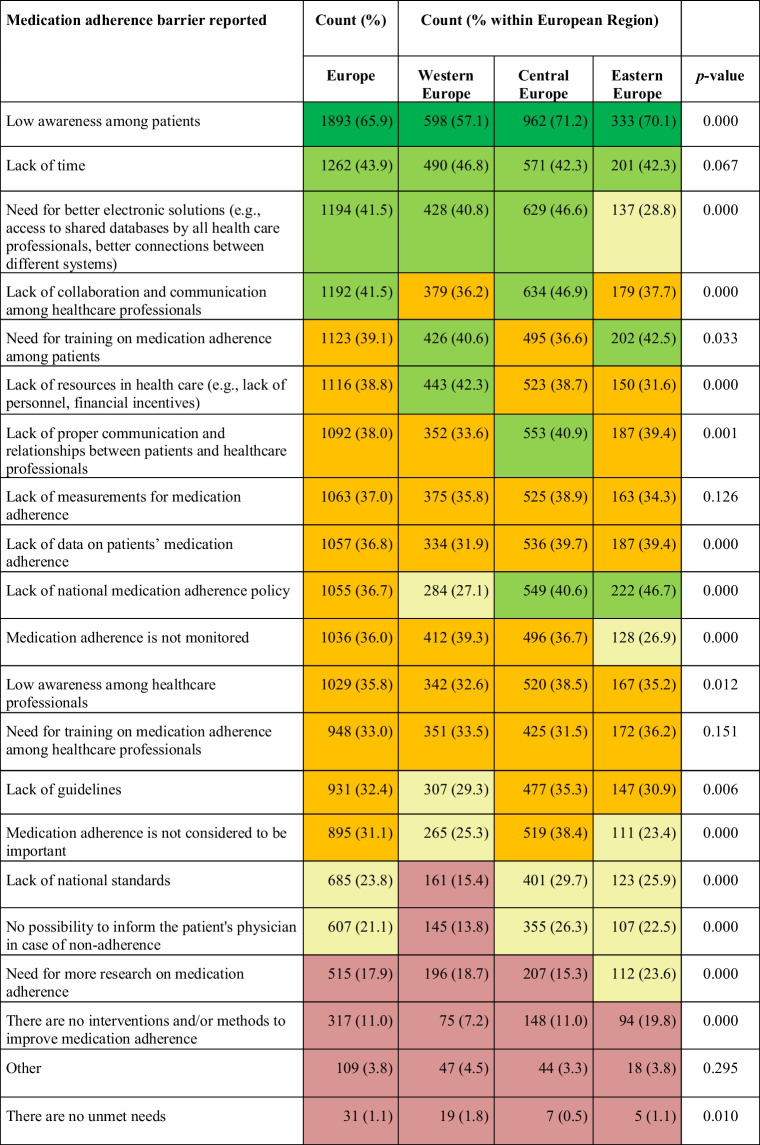
Healthcare professionals reported barriers regarding medication adherence management, stratified by European Regions. The color indicates the relative importance of the barrier (  : largest barrier (>50%);  : relatively large barrier (40–50%);  : medium barrier (30–40%);  : medium-low barrier (20–30%);  : relatively low barrier (<20%)). The data was obtained from the question “What would you identify as an unmet need regarding medication adherence in your daily work?”

Other overall relatively large health system barriers were “lack of time” (43.9%), “need for better electronic solutions” (e.g., access to shared databases and better connections between different databases) (41.5%), and “lack of collaboration and communication among HCPs” (41.5%). Some relevant regional differences were observed. For example, “lack of national MA policy” scored high as a health system barrier in Central and Eastern Europe (40.6% and 46.7% respectively) while scoring medium–low (27.1%) in Western Europe. Lack of collaboration and communication between HCPs was seen as a relatively high health system barrier in Central Europe particularly, but less in Western and Eastern Europe. Another regional difference was regarding the need for electronic solutions, which was prominent in Western and Central (40.8% and 46.6%, respectively) but only a medium–low perceived barrier in Eastern Europe (28.8%). Low-scoring barriers (i.e., not deemed a barrier) across all regions were lack of available research and interventions on MA.

**Fig. 1:**
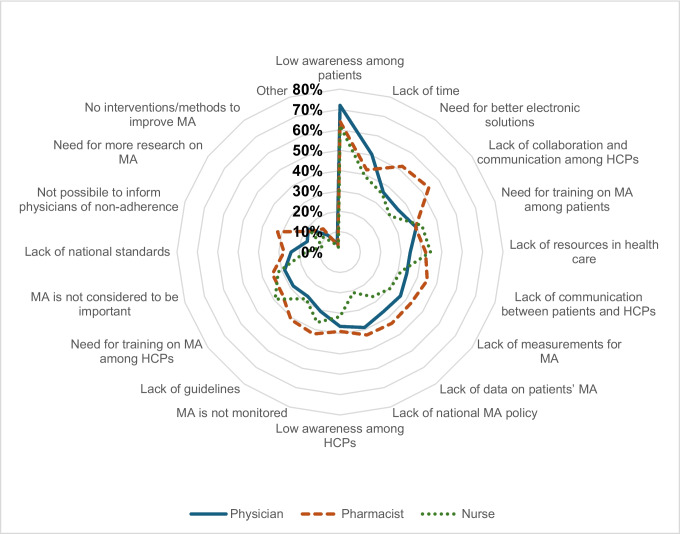
Unmet needs regarding medication adherence reported by European healthcare professionals, stratified by profession.

When stratified by health profession, some similarities but also some differences were observed (Supp Table S9, Fig. 1). Physicians, pharmacists, and nurses all ranked “low awareness among patients” as the largest barrier (62.2% for nurses to 72% for physicians). Physicians mentioned “lack of time” (50.4%) as another large barrier, while pharmacists mentioned “lack of collaboration and communication among HCPs” (53.9%) and “need for electronic solutions” (52%) as the second and third largest barriers. Finally, nurses mentioned “lack of resources” (44.4%) and “lack of training” (42.3%) as second and third largest barriers.

### HCP Training Needs Regarding MA Management

Almost all HCPs (98%) indicated the need for training in MA management (Table 3). Participants were able to choose more than one main training need, up to a maximum of three. Notably, “how to monitor and evaluate adherence” (35.9%) ranked as the highest need in Europe. “How to get patients to take an active role in their adherence management” (30.9%), “HCPs’ roles and responsibilities in adherence management” (30.4%), and “how to talk with patients about adherence” (26%) were deemed other overall high needs (Table 3).

There were some notable differences between regions. Western European HCPs referred the relatively highest need regarding “how to get the patient to take an active role in medication management” (35%), Central European HCPs scoring “HCPs’ roles and responsibilities” (35.8%) as the second highest need, and Eastern European HCPs indicating equally high needs (26–27%) regarding “HCPs’ roles and responsibilities,” “how to talk about adherence,” and “giving the patient an active role” (Table 3).

**Table 3.**
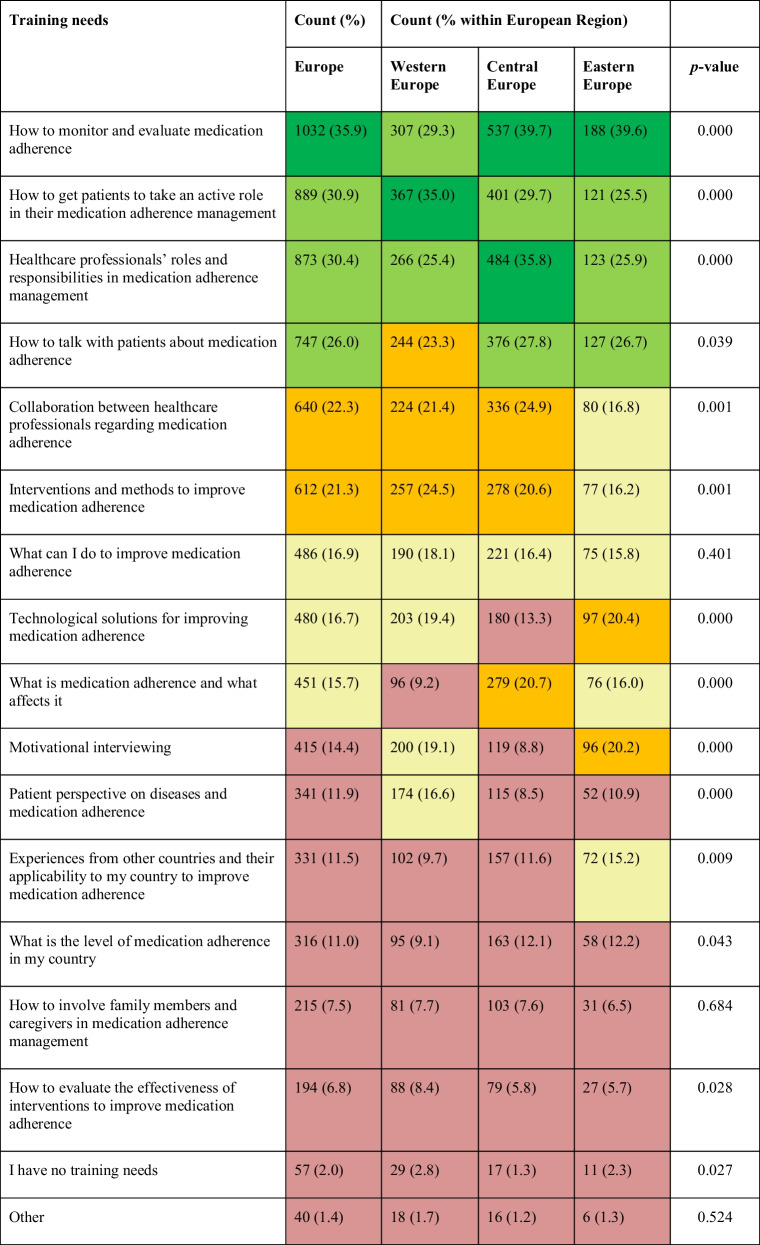
Healthcare professionals’ training needs regarding medication adherence management per European region (  : largest need (>35%);  : relatively large need (25–35%);  : medium need (20–25%);  : medium-low need (15–20%);  : relatively low need (<15%)). The data was obtained from the question “Please, identify the main training needs you have regarding medication adherence”

**Fig. 2:**
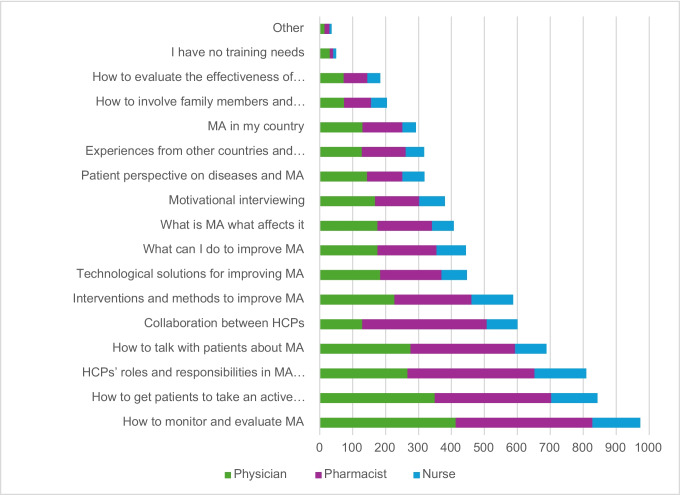
Training needs regarding medication adherence as reported by European healthcare professionals, stacked by profession (the figure represents the number of the responses within each profession).

Also, training needs showed some differences when stratified by profession, with for example the “need for collaboration between HCPs” more frequently mentioned by pharmacists (33%) compared to nurses (19.7%) and physicians (12.2%) (Supp Table S10, Fig. 2, Supp Figure S2).

## DISCUSSION

### Main Findings

The main perceived barriers to MA management by HCPs from 37 European countries are the lack of awareness among patients, a lack of time and resources, a lack of (access to shared) electronic monitoring databases and a lack of collaboration and communication among HCPs. Furthermore, almost all HCPs acknowledged a lack of training on the topic. The main training needs identified were adherence monitoring and assessment methods, how to actively involve patients in their treatment, and the clear definition of the roles and tasks of HCPs in MA management.

### Interpretation

This study highlights numerous barriers to adherence management in Europe, emphasizing a critical need for improvement. Lack of patient awareness was deemed the most important barrier, which underlines the need for continuous patient education. Of note, patients can decide to adjust, discontinue, or even not start medication at all, along the three phases of adherence that range from initiation, and implementation to persistence.^22,23^. Education on the importance of adequate adherence and managing expectations regarding potential adverse effects and costs is particularly essential during the first prescription as up to 15% do not even initiate newly prescribed medication.^24^ Still, follow-up education remains important given the chronic nature of many diseases for which medication is being prescribed. With an ageing population in Europe, and multi-morbidity and polypharmacy being more common during ageing, the risk of nonadherence also increases, and should therefore be considered in all available clinical practice guidelines for HCPs.^25,26^ Indeed, the need for more education is clear ^27^; however, the second barrier identified may complicate the achievement of improvement in this area given HCPs’ stressed lack of time and resources they are facing. Notably, given the duration of the survey administration, just after the COVID-19 pandemic, the impact of the pandemic, and further increasing HCP shortages may have influenced some of these answers.^28,29^ While requiring an initial investment, more time and resources allocated to feedback on patients’ medication use may be a cost-effective option in long term.^30^

The lack of electronic solutions seems to be a global trend that is rapidly shifting with a sharp increase in the development and availability of digital MA monitoring tools.^31,32^ Additionally, policy efforts on the European level, such as the European Health Data Space, are being made to address this issue.^33^ However, as promising this initiative seems, full implementation across Europe may require several more years. Access to a common electronic system may also solve the fourth large adherence barrier related to communication and collaboration between HCPs. Although it has been acknowledged that, e.g., physicians and pharmacists have their expertise regarding medications and their use ^34^, most of that knowledge is complimentary and could be more aligned and combined for the benefit of a clear and comprehensive message regarding optimal MA for the patient.

The observed notable differences between barriers by the European region and the healthcare profession deserve some comment. In Eastern Europe, HCPs were particularly concerned about the lack of national policies possibly linked to cultural factors or a lack of recognition of non-adherence by national professional bodies. Regarding professions, pharmacists identified the need for better electronic solutions and better communication between HCPs as prioritizing issues which aligns with their evolving role beyond dispensing.

Beyond the barriers they were facing, almost all HCPs expressed a strong need for training and education. Already in the early 1990s, the need for better education on how to address MA has been highlighted.^35^ Twenty years later, HCPs were still largely short of knowledge and insights on monitoring adherence and applying the appropriate interventions for individual patients.^18^ Another decade later, training needs still exist as reported in this study. Recent studies have demonstrated the educational impact, behavioral change in clinical practice, and improved health outcomes of structured training for HCPs.^36,37^. Training on monitoring and assessment methods was seen as the highest unmet training need, particularly in Central and Eastern Europe. Typically, different methods to measure adherence exist, each with advantages and disadvantages. Self-reported scales and electronic dispensing records are easy, quick, and cheap. Still, they are non-granular and may overestimate adherence, while digital technologies provide more granular data but are more expensive. Bioanalytical measures are the most objective but are invasive and provide only short-term adherence data.^2^

In current clinical European practice, patients are insufficiently empowered and lack tools to self-monitor, self-manage, and optimize drug use. This was especially prominent among Western European HCPs, who ranked it as the most pressing training need. Regarding the educational need for more involvement of patients in medication (self-)management, one important issue is HCPs’ awareness of patients’ health literacy for which specific training is available on clear and tailored communication techniques.^38^ Regarding communication practice with patients on adherence issues, other educational techniques of potential relevance include conversation analysis and simulated role plays.^39^ Additionally, considering regional European variations in barriers and needs, it is crucial to account for cultural differences when designing educational training programs, as emphasized in European medical education.^40^ The same may hold for other countries with cultural diversity (e.g., the USA).

### Strengths and Limitations

One of the main strengths of this study is its pan-European scope and the high number of respondents. The survey has been constructed with the utmost rigor and consensus among researchers from multiple countries and included an open-ended question pre-survey. Both surveys have been subjected to a pilot study for refinement and feasibility testing. Finally, to ensure broad participation, different language options for the survey were provided.

The study faced limitations, firstly in participant selection, leading to variations in representation across countries, professions, and sectors. Dependency on representatives resulted in uneven respondent numbers. The results were shown by profession and region to handle this problem. Despite unequal representation across countries, the most significant barriers were similar for most. Second, while the qualitative analysis ^41^ for the pre-survey followed several quality criteria, some investigator-level interpretations could have led to missing codes or misinterpretations. This was minimized by independent analysis and consensus building by two researchers for each code. Third, responder bias may exist, as those interested in adherence may be more inclined to participate.

ENABLE representatives’ recruitment methods might have influenced responder selection, hindering a response rate calculation due to unknown invitee numbers.

Initially, the pilot survey was in English, limiting responses to English speakers. To address this, respondents were allowed to reply in their native language during the pilot phase, reducing the need for extensive translation of the HCP survey into each country’s language.

### Implications

MA is critical for effective health management. Addressing the barriers, improving training, and fulfilling unmet needs in Europe and other parts of the world such as the USA are crucial for maintaining sustainable health systems. Our findings highlight the importance of addressing MA at different levels, from patient awareness to fostering collaboration among HCPs and implementing national policies. The identified training needs underscore the necessity of educating HCPs on various aspects of MA to enhance patient outcomes and healthcare practices. By acknowledging the cultural, socioeconomic, and healthcare system variations, and providing HCPs with the necessary training activities, we can collectively work towards improved adherence, better patient outcomes, and reduced healthcare costs. Indeed, proof of effective interventions ^42^ is available, but system barriers for implementation in daily clinical care such as providing sufficient time, resources, and infrastructure need to be properly addressed. Implementation of medication-enhancing activities requires intensified collaboration between HCPs at the regional level and stakeholders such as policymakers and insurers at the national level. Initiatives such as the European Health Data Space could offer further pan-European opportunities for common electronic health information systems.^33^ Such a common system could not only facilitate individual adherence support ^43^ but also data utilization used for targeted (artificial intelligence-informed) clinical decision support. Importantly, in doing so, patient concerns regarding data privacy and the impact of digital tools on the HCP-patient relationship should be carefully addressed.^44,45^ Notably, the WHO distinguishes between five different dimensions of MA: socioeconomic, health-system, condition-related, therapy-related, and patient-related non-adherence factors.^1^ This means that to fully tackle the non-adherence issue, addressing just health-system factors requires complementary actions on the four other levels as well.

## CONCLUSION

HCPs in Europe encounter barriers related to adequate MA management, with the most prominent barriers being the lack of patient awareness, lack of time, lack of suitable electronic solutions and shared prescription databases, and lack of collaboration and communication among HCPs. Training should primarily focus on adherence monitoring and management methods, defining HCPs’ responsibilities and tasks and how to stimulate patients’ active role in MA.

## Supplementary Information

Below is the link to the electronic supplementary material.

Supplementary file1 (DOCX 390 KB)

## Data Availability

The protocol and the study flow chart are available at 10.17605/OSF.IO/EBMZ5. The survey data is available on request from the corresponding author immediately following publication with no end date.
