# Supplementary material for: Barriers and Unmet Educational Needs Regarding Implementation of Medication Adherence Management Across Europe: Insights from COST Action ENABLE
Source: J Gen Intern Med. 2024 Jun 28;39(15):2917–26. doi: 10.1007/s11606-024-08851-2 (PMC11576669; doi:10.1007/s11606-024-08851-2)
Supplement: Supplementary file 1 — Supplementary file1 (DOCX 390 KB) [file 11606_2024_8851_MOESM1_ESM.docx]

**Appendix A. Supplementary data**

# Table S1. Checklist for Reporting Results of Internet E-Surveys (CHERRIES)

| No. | Item category | Checklist Item | Page Number |
| --- | --- | --- | --- |
| 1 | Design | Describe survey design | 5 |
| 2 | IRB (Institutional Review Board) approval and informed consent process | IRB approval | 6 |
|  |  | Informed consent | 6 |
|  |  | Data protection | 6 |
| 3 | Development and pre-testing | Development and testing | 5 |
| 4 | Recruitment process and description of the sample having access to the questionnaire | Open survey versus closed survey | 5 |
|  |  | Contact mode | 5 |
|  |  | Advertising the survey | 5 |
| 5 | Survey administration | Web/E-mail | 5 |
|  |  | Context | 5 |
|  |  | Mandatory/voluntary | 5 |
|  |  | Incentives | 5 |
|  |  | Time/Date | 5 |
|  |  | Randomisation of items or questionnaires | 5 |
|  |  | Adaptive questioning | 5 |
|  |  | Number of Items | 5 |
|  |  | Number of screens (pages) | - |
|  |  | Completeness check | 6 |
|  |  | Review step | Respondents were allowed to go 'back' in the survey. |
| 6 | Response rates | Unique site visitor | N/A |
|  |  | View rate (Ratio of unique survey visitors/unique site visitors) | The survey is voluntary. The system cannot record the number of unique visitors, so the view rate cannot be calculated. |
|  |  | Participation rate (Ratio of unique visitors who agreed to participate/unique first survey page visitors) | 6 |
|  |  | Completion rate (Ratio of users who finished the survey/users who agreed to participate) | 6 |
| 7 | Preventing multiple entries from the same individual | Cookies used | N/A |
|  |  | IP check | N/A |
|  |  | Log file analysis | N/A |
|  |  | Registration | N/A |
| 8 | Analysis | Handling of incomplete questionnaires | N/A |
|  |  | Questionnaires submitted with an atypical timestamp | N/A |
|  |  | Statistical correction | N/A |

**Table S2.** Structure of the questionnaire for medication adherence experts and methods of analysis.

| **Outcome** | **Variable*** | **Methods of Analysis** |
| --- | --- | --- |
| **i. Roles of different professions in the management of medication adherence** | 1. Which professions are involved in medication adherence in your country and what is their role in evaluating, monitoring, documenting, and/or improving adherence? | Percentages and frequencies for categories.  Segmentation and coding. |
| **ii. Existing and planned national initiatives and programs** | 7. Describe the existing and planned national policies, guidelines, frameworks, or campaigns on medication adherence in your country. If any documents of these policies etc are available, you can attach them after your answer or add an internet address where they can be found. | Answers will be read and re-read by 2 researchers independently, with subsequent triangulation. Segmentation of quotes and generation of codes to establish thematic groupings or categories |
|  | 10. Are there any specific initiatives or actions for improving adherence during the COVID-19 pandemic? If yes, please specify them. |  |
| **iii. Methods for analysis and type of adherence data collected** | 2. How can healthcare professionals evaluate patients' medication adherence in your country? |  |
|  | 3. What kind of patient-level data on medication adherence do healthcare professionals have access to? |  |
|  | 4. If a healthcare professional identifies a patient to be non-adherent, where can it be documented? |  |
| **iv. Barriers and unmet needs** | 8. What would you identify as an unmet need regarding medication adherence in your country? |  |
|  | 9. What barriers exist for medication adherence in your country (e.g., lack of resources, financing, activities, support)? |  |
| **v. Methods for adherence improvement** | 5. If a patient is identified as being non-adherent, what interventions can health professionals offer to improve adherence (for example, give consultation, organize closer follow-up, refer to an adherence intervention/program, mobile applications)? |  |
|  | 6. What would you identify as the most successful adherence interventions in your country within the interventions you mentioned in your answer to question 5? |  |

*order number in the questionnaire and question statement

**Table S3.** Medication adherence experts consulted per country

| **Country** | **Experts per country n (%)** |
| --- | --- |
| Austria | 2 (1.4) |
| Belgium | 2 (1.4) |
| Cyprus | 6 (4.3) |
| Finland | 4 (2.9) |
| France | 4 (2.9) |
| Germany | 3 (2.1) |
| Greece | 5 (3.6) |
| Iceland | 2 (1.4) |
| Ireland | 1 (0.7) |
| Italy | 4 (2.9) |
| Luxembourg | 1 (0.7) |
| Malta | 2 (1.4) |
| Netherlands | 4 (2.9) |
| Norway | 1 (0.7) |
| Portugal | 3 (2.1) |
| Spain | 5 (3.6) |
| Sweden | 5 (3.6) |
| Switzerland | 7 (5.0) |
| Albania | 2 (1.4) |
| Bosnia and Herzegovina | 5 (3.6) |
| Bulgaria | 5 (3.6) |
| Croatia | 4 (2.9) |
| Czechia | 2 (1.4) |
| Hungary | 5 (3.6) |
| North Macedonia | 5 (3.6) |
| Montenegro | 5 (3.6) |
| Poland | 8 (5.7) |
| Romania | 6 (4.3) |
| Serbia | 8 (5.7) |
| Slovakia | 5 (3.6) |
| Slovenia | 3 (2.1) |
| Estonia | 6 (4,3) |
| Latvia | 3 (2.1) |
| Lithuania | 3 (2.1) |
| Turkey | 4 (2.9) |
| **35 Countries** | **140 Respondents** |

**Table S4.** Working place of the medication adherence experts (n=140*) stratified by the European region.

| **Organisation** | **Europe** | **Western Europe** | **Central Europe** | **Eastern Europe** |
| --- | --- | --- | --- | --- |
|  | n (%) | n (%) | n (%) | n (%) |
| Research/academia | 74 (52.9) | 33 (54.1) | 32 (50.8) | 9 (56.3) |
| Hospital | 39 (27.9) | 21 (34.4) | 15 (23.8) | 3 (18.9) |
| Primary care | 27 (19.3) | 12 (19.7) | 10 (15.9) | 5 (31.3) |
| Community or hospital pharmacy | 24 (17.1) | 14 (30.0) | 7 (11.1) | 3 (18.9) |
| Private organisation/company | 11 (7.9) | 3 (4.9) | 8 (12.7) | 0 (0.0) |
| Professional association | 11 (7.9) | 4 (6.6) | 5 (7.9) | 2 (12.5) |
| Governmental | 9 (6.4) | 3 (4.9) | 5 (7.9) | 1 (6.3) |
| Pharmacoeconomics (e.g. consultancy) | 5 (3.6) | 1 (1.6) | 4 (6.3) | 0 (0.0) |
| Patient association | 5 (3.6) | 2 (3.3) | 2 (3.2) | 1 (6.3) |
| Insurance company | 1 (0.7) | 0 (0.0) | 1 (1.6) | 0 (0.0) |
| Other | 7 (5.0) | 4 (6.6) | 2 (3.2) | 1 (6.3) |

*Some experts were affiliated with multiple organisations

**Table S5.** Unmet needs and barriers regarding medication adherence per European Region according to medication adherence experts (n=140)

|  | **Europe  n (%)** | **Western Europe  n (%)** | **Central Europe  n (%)** | **Eastern Europe  n (%)** |
| --- | --- | --- | --- | --- |
| **LACK OF NATIONAL STRUCTURE AND SUPERORDINATE SUPPORT** | **67 (47.9)** | **23 (37.7)** | **32 (50.8)** | **12 (75.0)** |
| Lack of national guidelines and policies | 32 (22.9) | 10 (16.4) | 15 (23.8) | 7 (43.8) |
| Lack of coordination (upper level) | 18 (12.9) | 7 (11.5) | 8 (12.7) | 3 (18.8) |
| Lack of support for adherence management | 18 (12.9) | 10 (4,3) | 6 (9.5) | 2 (12.5) |
| No public campaigns & initiatives | 17 (12.1) | 4 (6.6) | 10 (15.9) | 3 (18.8) |
| **LACK OF AWARENESS AND LOW IMPORTANCE** | **48 (34.3)** | **23 (37.7)** | **17 (27.0)** | **8 (50.0)** |
| Insufficient recognition of the importance | 19 (13.6) | 7 (11.5) | 8 (12.7) | 4 (25.0) |
| Lack of awareness (general) | 14 (10.0) | 11 (18.0) | 3 (4.8) | 0 |
| Adherence is not a priority | 10 (7.1) | 3 (4.9) | 4 (6.3) | 3 (18.8) |
| Lack of awareness (HCPs) | 9 (6.4) | 7 (11.5) | 2 (3.2) | 0 |
| Lack of awareness (public/patients) | 4 (2.9) | 2 (3.3) | 1 (1.6) | 1 (6.3) |
| **LACK OF KNOWLEDGE AND EDUCATION** | **46 (32.9)** | **21 (34.4)** | **22 (34.9)** | **3 (18.8)** |
| Training of HCPs | 23 (16.4) | 13 (21.3) | 8 (12.7) | 2 (12.5) |
| Education (general) | 18 (12.9) | 9 (14.8) | 9 (14.3) | 0 |
| Patient education | 16 (11.4) | 3 (4.9) | 11 (17.5) | 2 (12.5) |
| **LIMITED HC RESOURCES** | **44 (31.4)** | **21 (34.4)** | **20 (31.7)** | **3 (18.8)** |
| Lack of time among HCPs | 20 (14.3) | 13 (21.3) | 6 (9.5) | 1 (6.3) |
| Lack of e-health solutions /IT systems | 17 (12.1) | 9 (14.8) | 7 (11.1) | 1 (6.3) |
| Lack of health care personnel | 17 (12.1) | 6 (9.8) | 10 (15.9) | 1 (6.3) |
| Lack of resources in healthcare | 9 (6.4) | 5 (8.2) | 3 (4.8) | 1 (6.3) |
| **ISSUES WITH THE IMPLEMENTATION OF ADHERENCE MANAGEMENT** | **28 (20.0)** | **14 (23.0)** | **12 (19.0)** | **2 (12.5)** |
| Lack of implementation of adherence management | 17 (12.1) | 7 (11.5) | 8 (12.7) | 2 (12.5) |
| Incentives/remuneration | 13 (9.3) | 7 (11.5) | 6 (9.5) | 0 |
| **ISSUES WITH ADHERENCE-ENHANCING INTERVENTIONS** | **26 (18.6)** | **15 (24.6)** | **9 (14.3)** | **2 (12.5)** |
| Lack of methods to improve adherence | 13 (9.3) | 6 (2,6) | 6 (9.5) | 1 (6.3) |
| Lack of e-health tools | 13 (9.3) | 9 (14.8) | 3 (4.8) | 1 (6.3) |
| Reimbursement of interventions | 3 (2.1) | 2 (3.3) | 1 (1.6) | 0 |
| **DETECTING NON-ADHERENCE** | **25 (17.9)** | **13 (21.3)** | **11 (17.5)** | **1 (6.3)** |
| Monitoring and evaluating adherence | 22 (15.7) | 12 (19.7) | 9 (14.3) | 1 (6.3) |
| Reporting of adherence | 5 (3.6) | 2 (3.3) | 3 (4.8) | 0 |
| **INTERACTION BETWEEN PATIENTS AND HCPS** | **25 (17.9)** | **13 (21.3)** | **10 (15.9)** | **2 (12.5)** |
| Patient-centred care | 17 (12.1) | 10 (16.4) | 7 (11.1) | 0 |
| Patient-HCP communication | 9 (6.4) | 3 (4.9) | 4 (6.3) | 2 (12.5) |
| **LACK OF WORKING TOGETHER TOWARDS A COMMON GOAL** | **23 (16.4)** | **11 (18.0)** | **11 (17.5)** | **1 (6.3)** |
| Fragmentation of healthcare | 18 (12.9) | 10 (16.4) | 7 (11.1) | 1 (6.3) |
| Communication between HCPs | 7 (5.0) | 1 (1.6) | 6 (9.5) | 0 |
| **ACCESS TO MEDICATIONS** | **18 (12.9)** | **8 (13.1)** | **7 (11.1)** | **3 (18.8)** |
| Medication costs | 17 (12.1) | 8 (13.1) | 7 (11.1) | 2 (12.5) |
| Other related to access | 5 (3.6) | 0 | 3 (4.8) | 2 (12.5) |
| **PATIENT PERSPECTIVE** | **12 (8.6)** | **3 (4.9)** | **7 (11.1)** | **2 (12.5)** |
| Patient beliefs and attitudes | 11 (7.9) | 3 (4.9) | 7 (11.1) | 1 (6.3) |
| Patient characteristics | 4 (2.9) | 2 (3.3) | 1 (1.6) | 1 (6.3) |
| **OTHER** | **75 (53.6)** | **28 (45.9)** | **37 (58.7)** | **10 (62.5)** |
| Lack of resources & activities (general) | 61 (43.6) | 18 (29.5) | 35 (55.6) | 8 (50.0) |
| Adherence research | 15 (10.7) | 9 (14.8) | 3 (4.8) | 3 (18.8) |
| Other | 5 (3.6) | 3 (4.9) | 1 (1.6) | 1 (6.3) |
| **NO BARRIERS** | **2 (1.4)** | **1 (1.6)** | **1 (1.6)** | **0** |

**Table S6.** Constructs included in the qualitative analysis and coding legend

| **Professions** | **Which professions are involved in medication adherence in your country and what is their role in evaluating, monitoring, documenting and/or improving adherence?** |
| --- | --- |
| C11 | Physicians/Doctors: Primary Care, Family doctors (GPs) and Medical Specialists, Clinical Pharmacologist |
| C12 | Pharmacists (community and hospital, clinical pharmacists) |
| C13 | Nurses |
| C14 | Dentists |
| C15 | Home Care Services |
| C16 | Psychologists |
| C17 | Other (Medical technicians and physiotherapists, midwifes, nursing aides, assistants, social services) |
| C18 | No profession involved |
| C19 | National health insurance institutes |
| C110 | Researchers |
| C199 | No information |
| **Barriers & unmet needs** | **What would you identify as an unmet need regarding medication adherence in your country? AND 9. What barriers exist to medication adherence in your country (e.g., lack of resources, financing, activities, support)?** |
| **LACK OF AWARENESS AND LOW IMPORTANCE** | |
| C81 | Lack of awareness (general) |
| C82 | Lack of awareness (HCPs) |
| C83 | Lack of awareness (public/patients) |
| C84 | Insufficient recognition of the importance |
| C85 | Adherence is not a priority |
| **LACK OF KNOWLEDGE AND EDUCATION** | |
| C86 | Patient education |
| C87 | Education (general) |
| C88 | Training of HCPs |
| **LACK OF NATIONAL STRUCTURE AND SUPERORDINATE SUPPORT** | |
| C89 | Lack of national guidelines and policies |
| C810 | Lack of coordination (upper level) |
| C811 | Public campaigns & initiatives |
| C812 | Lack of support for adherence management |
| **LACK OF WORKING TOGETHER TOWARDS A COMMON GOAL** | |
| C813 | Fragmentation of healthcare |
| C814 | Communication between HCPs |
| **ISSUES WITH ADHERENCE-ENHANCING INTERVENTIONS** | |
| C815 | Lack of methods to improve adherence |
| C816 | Lack of e-health tools |
| C817 | Reimbursement of interventions |
| **ISSUES WITH THE IMPLEMENTATION OF ADHERENCE MANAGEMENT** | |
| C818 | Incentives/remuneration |
| C819 | Lack of implementation of adherence management |
| **DETECTING NON-ADHERENCE** | |
| C820 | Monitoring and evaluating adherence |
| C821 | Reporting of adherence |
| **ACCESS TO MEDICATIONS** | |
| C822 | Medication costs |
| C823 | Other related to access |
| **PATIENT PERSPECTIVE** | |
| C824 | Patient beliefs and attitudes |
| C825 | Patient characteristics |
| **LIMITED HC RESOURCES** | |
| C826 | Lack of time among HCPs |
| C827 | Lack of e-health solutions /IT systems |
| C828 | Lack of health care personnel |
| C829 | Lack of resources in healthcare |
| **INTERACTION BETWEEN PATIENTS AND HCPS** | |
| C830 | Patient-HCP communication |
| C831 | Patient-centred care |
| **“ORPHAN” CODES** | |
| C832 | Adherence Research |
| C833 | No barriers |
| C834 | Other |
| C835 | Lack of resources & activities (general) |

**Table S7a.** Structure of the survey for healthcare professionals (HCPs)

| **Outcome** | **Variable** | **Methods of Analysis** |
| --- | --- | --- |
| **Profession, experience, and country** | What is your profession? | Percentages and frequencies for categories |
|  | Where do you encounter patients? If you have more than one workplace, please choose the one where you encounter issues related to medication adherence most often. |  |
|  | What kind of area is this workplace located in? |  |
|  | Which sector does this workplace represent? |  |
|  | What is your overall work experience? |  |
| **Monitoring medication adherence** | Do you monitor patients’ medication adherence regarding chronic medications?  How? Choose all that apply  Why not? Choose all that apply |  |
|  | If a patient is not reaching treatment goals, do you check his/her medication adherence? |  |
| **Reporting non-adherence** | If you identify a patient to be non-adherent, do you document or report it somewhere?  Where? Choose all that apply  Why not? Choose all that apply |  |
| **Improving medication adherence** | If you identify your patient to be non-adherent, do you try to improve his/her medication adherence?  How? Choose all that apply  Why not? Choose all that apply |  |
| **Unmet needs** | What would you identify as an unmet need regarding medication adherence in your daily work? Choose all that apply |  |
| **Training needs** | Please, identify the main training needs you have about medication adherence. You can choose a max of 3 options. |  |
|  | Would you like to attend training on medication adherence? |  |

**Table S7b.** Questions for the HCP survey

|  | Questions |
| --- | --- |
| Q1 | Informed consent |
| Q2 | Which country do you represent? |
| Q3 | What is your profession? |
| Q4 | Where do you encounter patients? If you have more than one workplace, please choose the one where you encounter issues related to medication adherence most often. |
| Q5 | What kind of area is this workplace located in? |
| Q6 | Which sector does this workplace represent? |
| Q7 | What is your overall work experience? |
| Q8 | Do you monitor patients’ medication adherence regarding chronic medications? |
| Q9 | How do you monitor your patients’ medication adherence? Choose all that apply |
| Q10 | Why don't you monitor medication adherence? Choose all that apply |
| Q11 | If you notice a patient is not reaching treatment goals, do you check his/her medication adherence? |
| Q12 | If you identify a patient to be non-adherent, do you document or report it somewhere? |
| Q13 | Where do you document non-adherence? Choose all that apply |
| Q14 | Why don’t you document non-adherence? Choose all that apply |
| Q15 | If you identify a patient to be non-adherent, do you try to improve his/her medication adherence? |
| Q16 | How do you try to improve your patient’s adherence? Choose all that apply |
| Q17 | Why don’t you try to improve your patients’ adherence? Choose all that apply |
| Q18 | What would you identify as an unmet need regarding medication adherence in your daily work? Choose all that apply |
| Q19 | Please, identify the main training needs you have regarding medication adherence. You can choose max 3 options. |
| *Additional* | Would you like to attend training on medication adherence? |

**Table S8.** Healthcare professionals responding to the survey, per country (n=37) according to the European Region

|  | **Country** | **n** | **Percent** |
| --- | --- | --- | --- |
| WESTERN EUROPE | Austria | 3 | 0.1% |
|  | Belgium | 2 | 0.1% |
|  | Cyprus | 82 | 2.8% |
|  | Finland | 228 | 7.9% |
|  | France | 83 | 2.9% |
|  | Germany | 5 | 0.2% |
|  | Greece | 31 | 1.1% |
|  | Iceland | 36 | 1.3% |
|  | Ireland | 2 | 0.1% |
|  | Israel | 12 | 0.4% |
|  | Italy | 5 | 0.2% |
|  | Luxembourg | 1 | 0.0% |
|  | Malta | 2 | 0.1% |
|  | Netherlands | 58 | 2.0% |
|  | Norway | 1 | 0.0% |
|  | Portugal | 52 | 1.8% |
|  | Spain | 165 | 5.7% |
|  | Sweden | 51 | 1.8% |
|  | Switzerland | 99 | 3.4% |
|  | United Kingdom | 131 | 4.6% |
| Subtotal |  | 1049 | 36.5% |
| CENTRAL EUROPE | Albania | 50 | 1.7% |
|  | Bosnia and Herzegovina | 70 | 2.4% |
|  | Bulgaria | 101 | 3.5% |
|  | Croatia | 285 | 9.9% |
|  | Hungary | 6 | 0.2% |
|  | Republic of North Macedonia | 58 | 2.0% |
|  | Montenegro | 8 | 0.3% |
|  | Poland | 42 | 1.5% |
|  | Romania | 432 | 15.0% |
|  | Serbia | 104 | 3.6% |
|  | Slovakia | 15 | 0.5% |
|  | Slovenia | 180 | 6.3% |
| Subtotal |  | 1351 | 47.0% |
| EASTERN EUROPE | Estonia | 139 | 4.8% |
|  | Latvia | 5 | 0.2% |
|  | Lithuania | 84 | 2.9% |
|  | Turkey | 150 | 5.2% |
|  | Ukraine | 97 | 3.4% |
| Subtotal |  | 475 | 16.5% |
| TOTAL |  | 2875 | 100.0% |

*The survey was translated into 24 languages: English- Finnish, French, German, Greek, Icelandic, Italian, Dutch, Portuguese, Spanish, Swedish, Albanian, Bosnian, Bulgarian, Croatian, Polish, Romanian, Serbian, Slovak, Slovenian, Estonian, Latvian, Lithuanian, Turkish and Czech. Since there was no survey participant from the Czech Republic, it is not shown in this table.

**Table S9.** Reported barriers regarding medication adherence management by European healthcare professionals, stratified by profession (dark green: largest barrier (>50%), green: relatively large barrier (40-50%), orange: medium barrier (30-40%), yellow: medium-low barrier (20-30%), pink: relatively low barrier (<20%)) The data was obtained from the question “What would you identify as an unmet need regarding medication adherence in your daily work?”

| **Count (% within the profession)** | **Total** | **Physician** | **Pharmacist** | **Nurse** |
| --- | --- | --- | --- | --- |
| Low awareness among patients | 1790 (66.9) | 760 (72.0) | 736 (64.2) | 294 (62.2) |
| Lack of time | 1205 (45.0) | 532 (50.4) | 487 (42.5) | 186 (39.3) |
| Need for better electronic solutions (e.g., access to shared databases by all health care professionals, better connections between different | 1146 (42.8) | 382 (36.2) | 597 (52.0) | 167 (35.3) |
| Lack of collaboration and communication among healthcare professionals | 1133 (42.4) | 372 (35.3) | 618 (53.9) | 143 (30.2) |
| Need for training on medication adherence among patients | 1062 (39.7) | 415 (39.3) | 447 (39.0) | 200 (42.3) |
| Lack of resources in health care (e.g., lack of personnel, financial incentives) | 1056 (39.5) | 364 (34.5) | 482 (42.0) | 210 (44.4) |
| Lack of proper communication and relationships between patients and healthcare professionals | 1027 (38.4) | 363 (34.4) | 516 (45.0) | 148 (31.3) |
| Lack of measurements for medication adherence | 1023 (38.2) | 387 (36.7) | 492 (42.9) | 144 (30.4) |
| Lack of data on patients’ medication adherence | 1006 (37.6) | 382 (36.2) | 496 (43.2) | 128 (27.1) |
| Lack of national medication adherence policy | 1001 (37.4) | 411 (39.0) | 491 (42.8) | 99 (20.9) |
| Low awareness among healthcare professionals | 982 (36.7) | 385 (36.5) | 447 (39.0) | 150 (31.7) |
| Medication adherence is not monitored | 981 (36.7) | 324 (30.7) | 485 (42.3) | 172 (36.4) |
| Lack of guidelines | 888 (33.2) | 284 (26.9) | 470 (41.0) | 134 (28.3) |
| Need for training on medication adherence among health care professionals | 887 (33.2) | 299 (28.3) | 402 (35.0) | 186 (39.3) |
| Medication adherence is not considered to be important | 852 (31.9) | 301 (28.5) | 400 (34.9) | 151 (31.9) |
| Lack of national standards | 648 (24.2) | 253 (24.0) | 315 (27.5) | 80 (16.9) |
| No possibility to inform the patient's physician in case of non-adherence | 589 (22.0) | 178 (16.9) | 369 (32.2) | 42 (8.9) |
| Need for more research on medication adherence | 488 (18.2) | 203 (19.2) | 198 (17.3) | 87 (18.4) |
| There are no interventions and/or methods to improve medication adherence | 297 (11.1) | 109 (10.3) | 161 (14.0) | 27 (5.7) |
| Other | 95 (3.6) | 46 (4.4) | 37 (3.2) | 12 (2.5) |
| There are no unmet needs | 28 (1.0) | 17 (1.6) | 4 (0.3) | 7 (1.5) |

**Table S10.** Training needs regarding medication adherence management reported by European healthcare professionals, stratified by profession (dark green: largest need (>35%), green: relatively large need (25-35%), orange: medium need (20-25%), yellow: medium-low need (15-20%), pink: relatively low need (<15%)) The data was obtained from the question “Please, identify the main training needs you have regarding medication adherence”

| **Count (% within Profession)** | **Total** | **Physician** | **Pharmacist** | **Nurse** |
| --- | --- | --- | --- | --- |
| How to monitor and evaluate medication adherence | 973 (36.4) | 413 (39.1) | 415 (36.2) | 145 (30.7) |
| How to get patients to take an active role in their medication adherence management | 843 (31.5) | 349 (33.1) | 354 (30.9) | 140 (29.6) |
| Healthcare professionals’ roles and responsibilities in medication adherence management | 809 (30.2) | 267 (25.3) | 385 (33.6) | 157 (33.2) |
| How to talk with patients about medication adherence | 688 (25.7) | 276 (26.2) | 317 (27.6) | 95 (20.1) |
| Collaboration between healthcare professionals regarding medication adherence | 600 (22.4) | 129 (12.2) | 378 (33.0) | 93 (19.7) |
| Interventions and methods to improve medication adherence | 587 (21.9) | 227 (21.5) | 234 (20.4) | 126 (26.6) |
| Technological solutions for improving medication adherence | 447 (16.7) | 184 (17.4) | 186 (16.2) | 77 (16.3) |
| What can I do to improve medication adherence | 444 (16.6) | 176 (16.7) | 179 (15.6) | 89 (18.8) |
| What is medication adherence and what affects it | 407 (15.2) | 176 (16.7) | 165 (14.4) | 66 (14.0) |
| Motivational interviewing | 380 (14.2) | 168 (15.9) | 134 (11.7) | 78 (16.5) |
| Patient perspective on diseases and medication adherence | 318 (11.9) | 144 (13.6) | 107 (9.3) | 67 (14.2) |
| Experiences from other countries and their applicability to my country to improve medication adherence | 317 (11.9) | 128 (12.1) | 133 (11.6) | 56 (11.8) |
| What is the level of medication adherence in my country | 292 (10.9) | 130 (12.3) | 121 (10.5) | 41 (8.7) |
| How to involve family members and caregivers in medication adherence management | 204 (7.6) | 74 (7.0) | 82 (7.1) | 48 (10.1) |
| How to evaluate the effectiveness of interventions to improve medication adherence | 184 (6.9) | 73 (6.9) | 72 (6.3) | 39 (8.2) |
| I have no training needs | 50 (1.9) | 31 (2.9) | 10 (0.9) | (1.9) |
| Other | 36 (1.3) | 15 (1.4) | 15 (1.3) | 6 (1.3) |


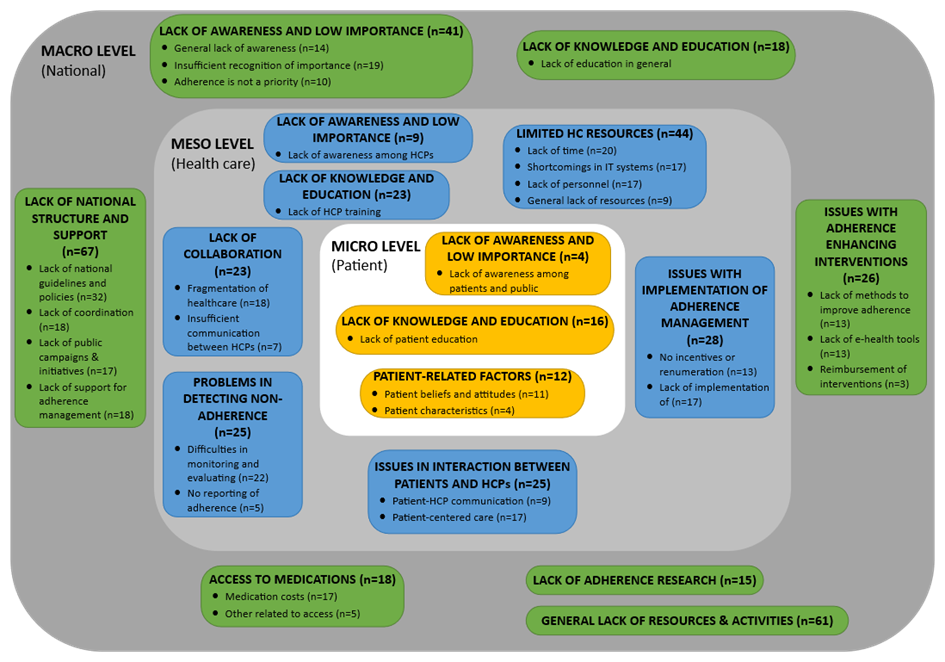


**Figure S1** Medication adherence expert study framework on unmet needs and barriers to medication adherence


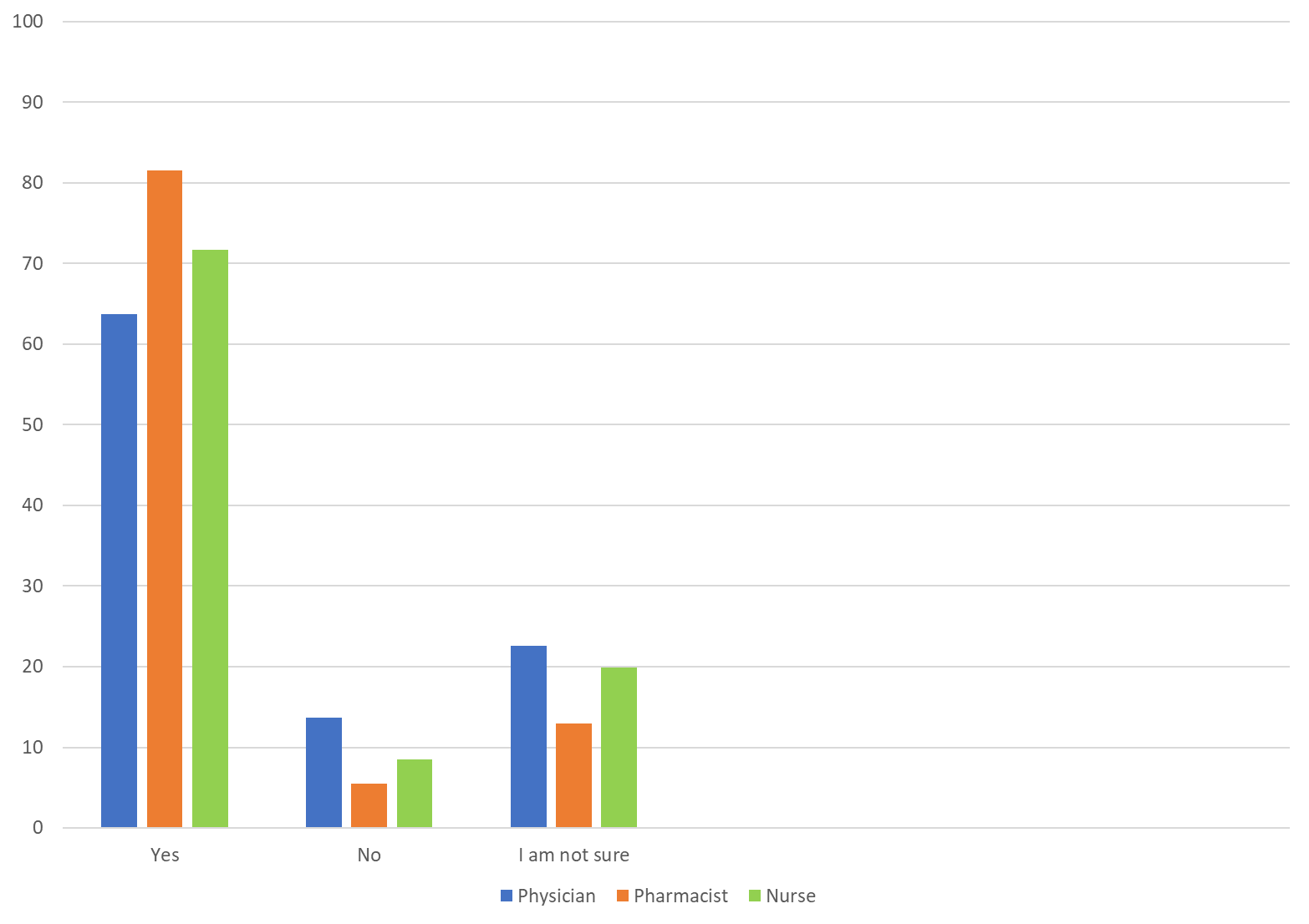

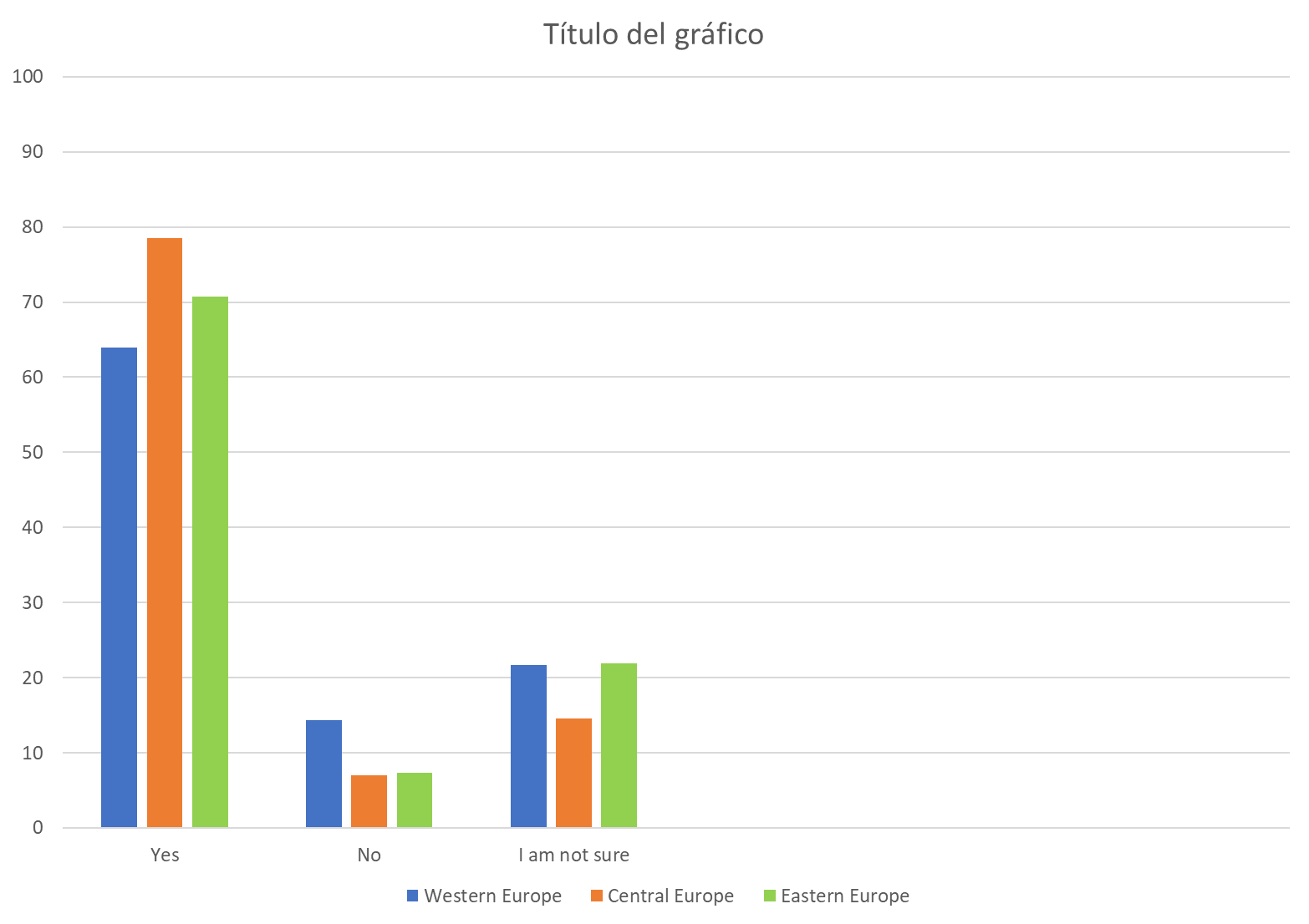


**Figure S2.** Healthcare Professionals (HCPs) Survey: Intention to attend training activities on medication adherence by European Region and profession
